# Comprehensive analysis of prognostic immune-related genes in the tumor microenvironment of colorectal cancer

**DOI:** 10.18632/aging.202479

**Published:** 2021-02-01

**Authors:** Wei Li, Xiaojing Jin, Shang Guo, Fei Xu, Xingkai Su, Xia Jiang, Guiqi Wang

**Affiliations:** 1 Department of General Surgery, Hebei Key Laboratory of Colorectal Cancer Precision Diagnosis and Treatment, The First Hospital of Hebei Medical University, Shijiazhuang, Hebei, China; 2 Department of Emergency Medicine, The First Hospital of Hebei Medical University, Shijiazhuang, Hebei, China

**Keywords:** colorectal cancer, TCGA, GEO, prognosis, nomogram

## Abstract

In this study, we used the ESTIMATE algorithm to analyze clinical data and transcriptome profiles of 1635 colorectal cancer (CRC) samples from the Gene Expression Omnibus and The Cancer Genome Atlas databases and identify prognostic immune-related genes (IRGs). We identified 941 differentially expressed (4 downregulated and 937 upregulated) genes by comparing samples with high and low immune, stromal scores and tumor purity. LASSO Cox regression analyses showed that the risk score based on a ten-IRG signature was an independent prognostic factor in CRC. The nomogram with pathological stages (TNM) and the ten-IRG signature showed a C-index of 0.769 (95% CI, 0.717-0.821), and area under ROC curve values of 0.788, 0.782 and 0.789 for 1-, 3-, and 5-year OS, respectively. TIMER database analysis showed positive correlation between the ten prognostic IRGs and the levels of tumor-infiltrated immune cells, including CD4^+^ and CD8^+^ T cells, macrophages, neutrophils, and dendritic cells. These findings demonstrate that this novel ten-IRG signature correlates with the pathological stages and the levels of multiple tumor-infiltrated immune cell types. This makes the ten-IRG signature a potential prognostic factor for CRC patients.

## INTRODUCTION

Colorectal cancer (CRC) is the third most commonly diagnosed cancer among men and women, and the second leading cause of cancer deaths worldwide [1, 2]. The tumor microenvironment (TME) plays a crucial role in tumor development and progression and consists of cancer cells, stromal cells, and several types of immune cells [3, 4]. Colorectal cancer is highly immunogenic and may show positive response to immunotherapy [5–7]. In several cancers, normalization of the tumor microenvironment improves the efficacy of targeted therapies, radiotherapy, and chemotherapy [8–10]. This suggests that the proportion of different immune cell types and their functional status in the TME determines the efficacy of immunotherapy in CRC.

Several studies have demonstrated the association between TME and survival outcomes as well as tumor recurrence in CRC patients. Xiong et al. showed that reduced levels of M1 macrophages and elevated levels of M2 macrophages, neutrophils, and eosinophils in the TME were associated with poor prognosis of CRC patients [11]. Furthermore, Ye et al. reported significant correlation between the levels of Tregs, neutrophils, and macrophages in the TME and the prognosis of CRC patients [12]. Recent advances in high-throughput sequencing technologies have helped define the genomic landscape of CRC and identify several TME-related molecular signatures for predicting the prognosis of CRC patients [13–15]. However, these molecular signatures require further analysis and validation to explore their utility in clinical applications.

Large-scale transcriptome, genomic, mutational, and clinical data of cancer patients is now available from public databases like Gene Expression Omnibus (GEO) and The Cancer Genome Atlas (TCGA). Yoshihara et al. developed a novel algorithm to called ESTIMATE (Estimation of Stromal and Immune cells in Malignant Tumors using Expression data) to compute the fraction of stromal and immune cells in tumor tissues [16]. ESTIMATE algorithm generates three scores-stromal, immune, and estimate. Stromal and immune scores are a measure of the proportion of stromal cells and immune cells in the tumor tissues, respectively. Estimate score indicates tumor purity, which is defined as the percentage of tumor cells in the TME and is closely related to the prognosis of cancer. Recent studies have shown that ESTIMATE scores show the extent of infiltration of non-tumor cells such as the immune cell types into the TME of cutaneous melanoma, glioblastoma, and adrenocortical carcinoma [17–19].

In this study, we analyzed the transcriptome data of CRC patients from the TCGA and GEO databases using the ESTIMATE algorithm to identify and characterize immune-related genes (IRGs) that can be used to accurately predict the prognosis of CRC patients. Furthermore, we analyzed the potential prognostic IRGs using the LASSO (Least Absolute Shrinkage and Selection Operator) Cox regression model and We constructed a nomogram with ten prognostic IRGs after analysis using the LASSO Cox regression model and further evaluated the correlation between clinicopathological features and the expression status of the prognostic IRGs in CRC samples.

## RESULTS

### ESTIMATE analysis shows relationship between tumor purity, stromal scores, and immune scores with CRC prognosis

**Figure 1:**
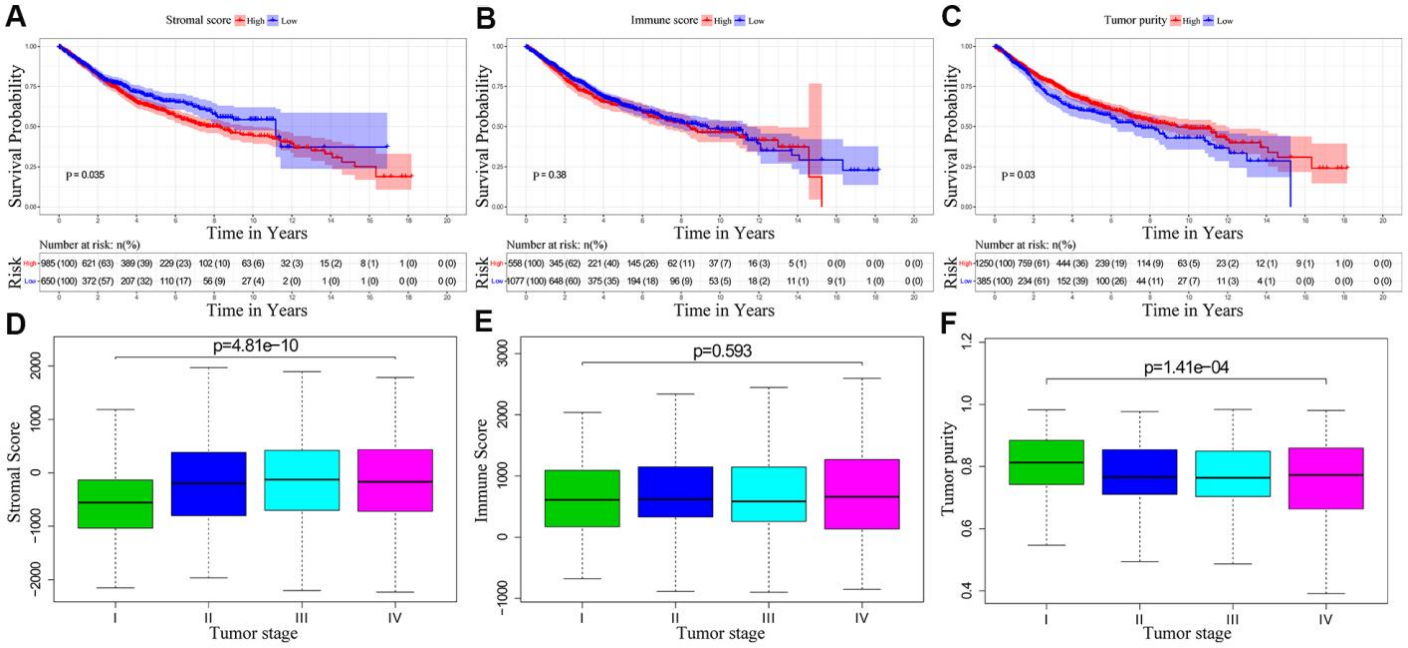
**The relationship between stromal score, immune score and tumor purity of CRC patient samples with pathological stages and overall survival.** (**A**) Kaplan-Meier survival curve analysis shows overall survival of CRC patients with high and low stromal scores. (**B**) Kaplan-Meier survival curve analysis shows overall survival of CRC patients with high and low immune scores. (**C**) Kaplan-Meier survival curve analysis shows overall survival of CRC patients with high and low tumor purity. (**D**) Correlation analysis between stromal scores and pathological stages of CRC patients. (**E**) Correlation analysis between immune scores and pathological stages of CRC patients. (**F**) Correlation analysis between tumor purity and pathological stages of CRC patients.

We downloaded OS and clinicopathological data for 1635 eligible CRC patients from the TCGA and GEO databases with a mean age of 68 years at diagnosis. Among these, 53.9% of the CRC patients were males. ESTIMATE results showed that immune scores ranged from -899.57 and 3202.84, stromal scores ranged from -2232.54 to 2193.08, and tumor purity ranged from 0.27 to 0.98. We then classified the CRC patients into low- and high-score groups based on their immune, stromal, and tumor purity scores, and investigated their correlation with OS rates. We observed positive correlation between stromal scores and OS of CRC patients (p=0.035; Figure 1A). However, immune scores did not show any significant correlation with OS of CRC patients (p=0.381; Figure 1B). We also observed negative correlation between tumor purity and OS (p=0.03; Figure 1C). We then assessed the relationship between immune scores, stromal scores and tumor purity with the pathological stages of CRC. We observed positive correlation between stromal scores and pathologic stage (p = 4.815e-10; Figure 1D). Immune scores were not associated with the pathological stage of CRC patients (p = 0.593; Figure 1E). Tumor purity showed inverse correlation with the pathological stages of CRC patients (p = 1.411e-04; Figure 1F).

### Identification of differentially expressed immune- related genes in CRC tissues

**Figure 2:**
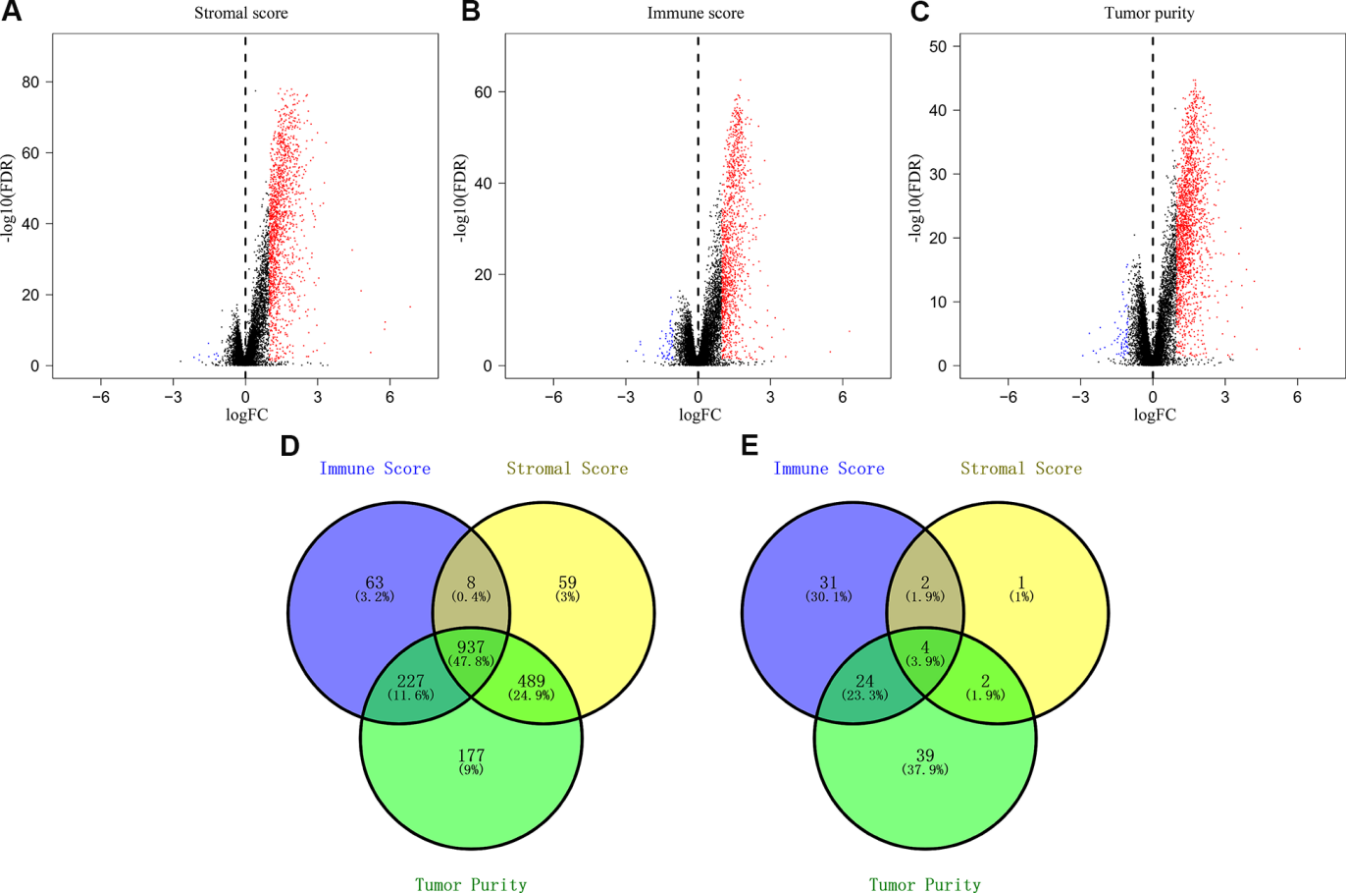
**Identification of differentially expressed genes based on the immune scores, stromal scores, and tumor purity of CRC patients.** (**A**) Volcano plots of the DEGs based on stromal scores. (**B**) Volcano plots of the DEGs based on immune scores. (**C**) Volcano plots of the DEGs based on tumor purity. (**D**) Venn diagram shows the numbers of upregulated genes in the immune score, stromal score and tumor purity groups as well as the upregulated genes that are common among the three groups. (**E**) Venn diagram shows the numbers of downregulated genes in immune score, stromal score and tumor purity groups as well as the downregulated genes that are common among the three groups.

As shown by the volcano plots, we identified several differentially expressed genes (upregulated and downregulated) between the low- and high-score groups in the TCGA-CRC cohort (n=611; Figure 2A–2C). We identified 9 downregulated and 1493 upregulated genes in the high stromal score group compared to the low stromal score group (Figure 2A), 61 downregulated and 1235 upregulated genes in the high immune score group compared to the low immune score group (Figure 2B), and 1830 upregulated genes and 69 downregulated genes in the low tumor purity group compared to the high tumor purity score group (Figure 2C). Overall, we identified 4 downregulated and 937 upregulated IRGs that were common among all the three groups (Figure 2D–2E).

### Functional enrichment analysis of the differentially expressed IRGs

**Figure 3:**
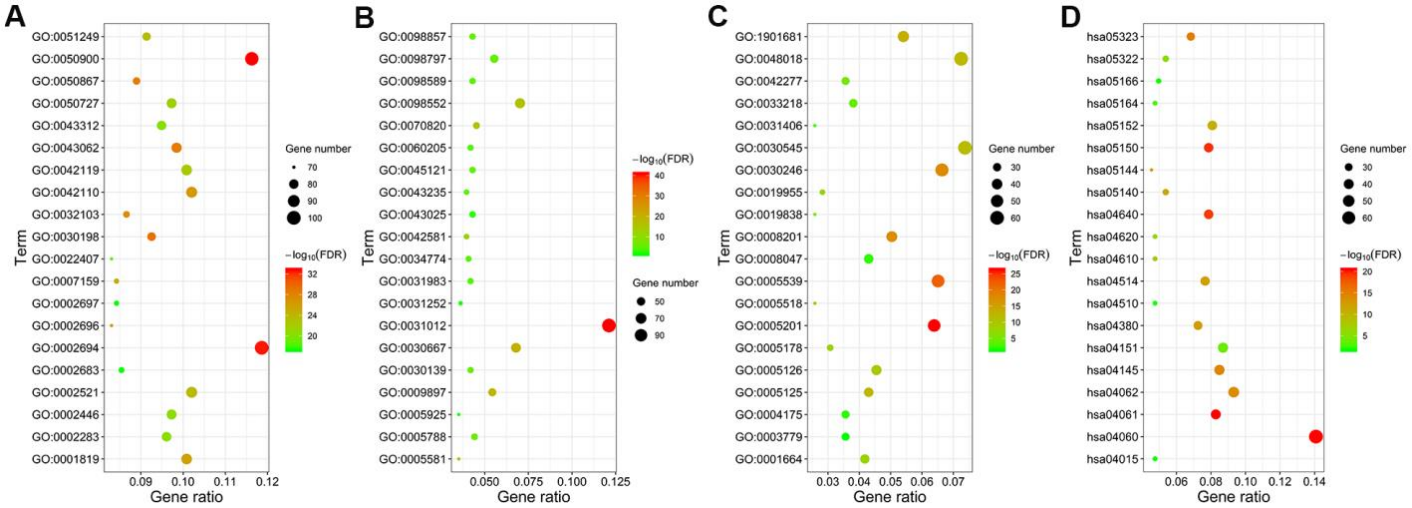
**Functional enrichment analysis of the differentially expressed IRGs.** GO and KEGG pathway analyses results show the most enriched GO terms related to (**A**) biological functions (BF), (**B**) cellular component (CC), and (**C**) molecular functions (MF), and (**D**) KEGG pathways related to differentially expressed IRGs.

We performed functional enrichment analysis using the clusterProfiler R package and identified 1454 significantly enriched GO terms and 54 significantly enriched KEGG pathways (FDR<0.05) associated with the 941 differentially expressed IRGs. The top 20 GO terms and KEGG pathways are shown in Figure 3 and Supplementary Table 1. The top GO terms related to biological processes (BF) included regulation of leukocyte activation (GO:0002694), leukocyte migration (GO:0050900), and T cell activation (GO:0042110). The top GO terms related to cellular components (CC) were membrane (GO:0098552) and extracellular matrix (GO:0031012). The top GO terms related to molecular functions (MF) included receptor regulator activity (GO:0030545) and receptor ligand activity (GO:0048018). The top enriched KEGG pathways were cytokine-cytokine receptor interactions (hsa04060), chemokine signaling pathway (hsa04062), PI3K-Akt signaling pathway (hsa04151), and phagosomes (hsa04145).

### Construction and validation of the IRG-based prognostic signature

**Figure 4:**
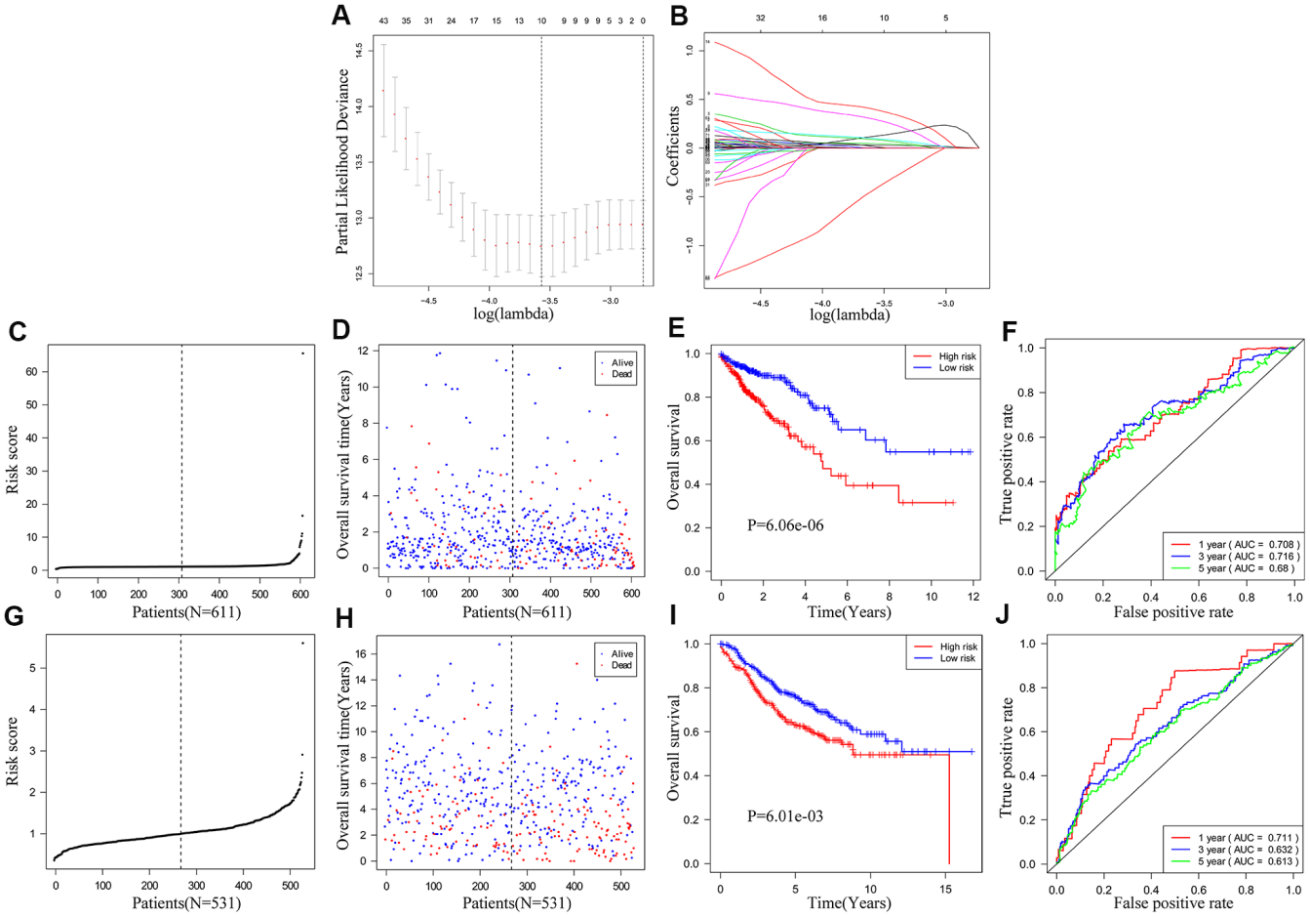
**Construction and validation of the ten-IRG prognostic signature.** (**A**) A plot of partial likelihood deviance of the LASSO coefficient profiles of differentially expressed IRGs. (**B**) A plot of the LASSO coefficient profiles for the differentially expressed IRGs associated with overall survival of CRC. (**C**) Distribution of risk scores for the CRC patients in the TCGA cohort based on the ten-IRG prognostic signature. (**D**) The survival status of 611 CRC patients in the TCGA cohort belonging to the high- and low-risk groups based on the ten-IRG prognostic signature. (**E**) Kaplan–Meier survival curves show overall survival of high and low-risk CRC patients in the TCGA cohort based on the ten-IRG prognostic signature. (**F**) Time-dependent ROC curves show the accuracy of overall survival prediction for the TCGA-CRC cohort based on the ten-IRG prognostic signature. (**G**) Distribution of risk scores for the GSE39582 cohort based on the ten-IRG prognostic signature. (**H**) The survival status of 531 CRC patients in the GSE3958 cohort belonging to the high- and low-risk groups based on the ten-IRG prognostic signature. (**I**) Kaplan–Meier survival curve analysis shows the overall survival of high and low-risk CRC patients in the GSE3958 cohort based on the ten-IRG prognostic signature. (**J**) Time-dependent ROC curves show the accuracy of overall survival prediction for the GSE3958 cohort based on the ten-IRG prognostic signature.

We used univariate COX regression and Lasso regression analysis to identify IRGs that predict the prognosis of CRC patients. We identified 172 IRGs based on the LASSO regression analysis results that were significantly correlated with the OS of CRC patients (Figure 4A, 4B). Subsequently, based on the co-efficient values, we generated an IRG-based prognostic risk signature with ten IRGs. The risk score was calculated with the following formula: (0.1235 × expression of MAP2) + (0.0873 × expression of NKAIN4) + (0.2936 × expression of VAX2 status) + (0.0321 × expression of CALB2) + (0.3958 × expression of FCRL2) + (0.0471 × expression of HAND1) + (0.0986 × expression of A2ML1) + (0.002 × expression of IDO1) + (0.0134 × expression of COL22A1) + (-0.4143 × expression of CD1B). CRC patients from the TCGA database (n=611) were then classified into low- and high-risk groups according to the cutoff risk score of 1.099 (Figure 4C, 4D). The high-risk group CRC patients showed significantly lower OS than those in the low-risk group (p < 0.0001; Figure 4E). The area under the ROC curve (AUC) values for 1-, 3-, and 5-year OS were 0.708, 0.716, and 0.680, respectively (Figure 4F). In the internal validation cohort, patients from the high-risk group demonstrated significantly worse survival outcomes compared to the low-risk group (P<0.001; Supplementary Figure 1). The AUC values for the internal validation cohort were 0.722, 0.712, and 0.738, for 1-, 3- and 5-year OS, respectively (Supplementary Figure 1). Next, we verified the prognostic model in the GSE39582 cohort (n=531) by first classifying the CRC patients into low- and high-risk groups according to the cutoff risk score of 0.989 (Figure 4G, 4H). The high-risk CRC patients from the GSE39582 cohort showed significantly lower OS than the corresponding low-risk patients (p = 0.006; Figure 4I). The AUC values for 1-, 3-, and 5-year OS for CRC patients from the GSE39582 cohort were 0.711, 0.632, and 0.613, respectively (Figure 4J).

### The ten-IRG signature is an independent prognostic predictor for CRC patients

**Figure 5:**
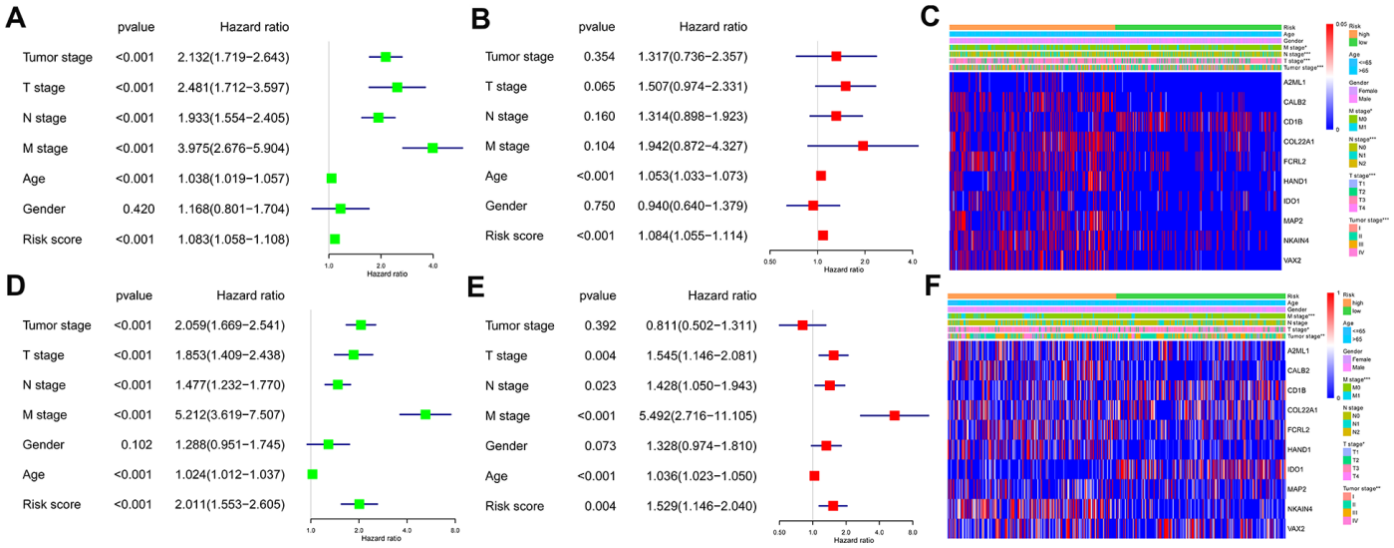
**Association of the ten-IRG prognostic signature with overall survival of CRC patients.** (**A**) Univariate COX regression analysis shows the clinicopathological parameters associated with the overall survival of CRC patients in the TCGA cohort. (**B**) Multivariate COX regression analysis shows clinicopathological parameters associated with the overall survival of CRC patients in the TCGA cohort. (**C**) Correlation analysis results show the relationship between the ten-IRG prognostic signature and the clinicopathological parameters in the TCGA-CRC cohort. (**D**) Univariate COX regression analysis shows the clinicopathological parameters associated with the overall survival of CRC patients in the GSE39582 cohort. (**E**) Multivariate COX regression analysis shows clinicopathological parameters associated with the overall survival of CRC patients in the GSE39582 cohort. (**F**) Correlation analysis results show the relationship between the ten-IRG prognostic signature and the clinicopathological parameters in the GSE39582-CRC cohort.

We performed univariate and multivariate Cox regression analyses using the TCGA and GEO CRC patient datasets after adjusting for clinicopathological parameters such as M stage, N stage, T stage, tumor stage, age and gender to determine if the risk score based on the ten-IRG signature accurately predicted prognosis of CRC patients. The results showed that the ten-IRG signature-based risk score was an independent prognosis factor for determining the OS of CRC patients from the two datasets (Figure 5A, 5B, 5D, 5E and Supplementary Figure 1). Next, we analyzed the correlation between the ten-IRG signature and other clinicopathological parameters using the Chi-square test. In the TCGA cohort, the higher risk score based on the ten-IRG signature was associated with tumor stage (p<0.001), M stage (p<0.05), N stage (p<0.001), and T stage (p<0.001) (Figure 5C), but was not related to gender and age of CRC patients. We obtained similar results with the GSE39582 validation cohort of CRC patients (Figure 5F).

### Nomogram construction and validation

**Figure 6:**
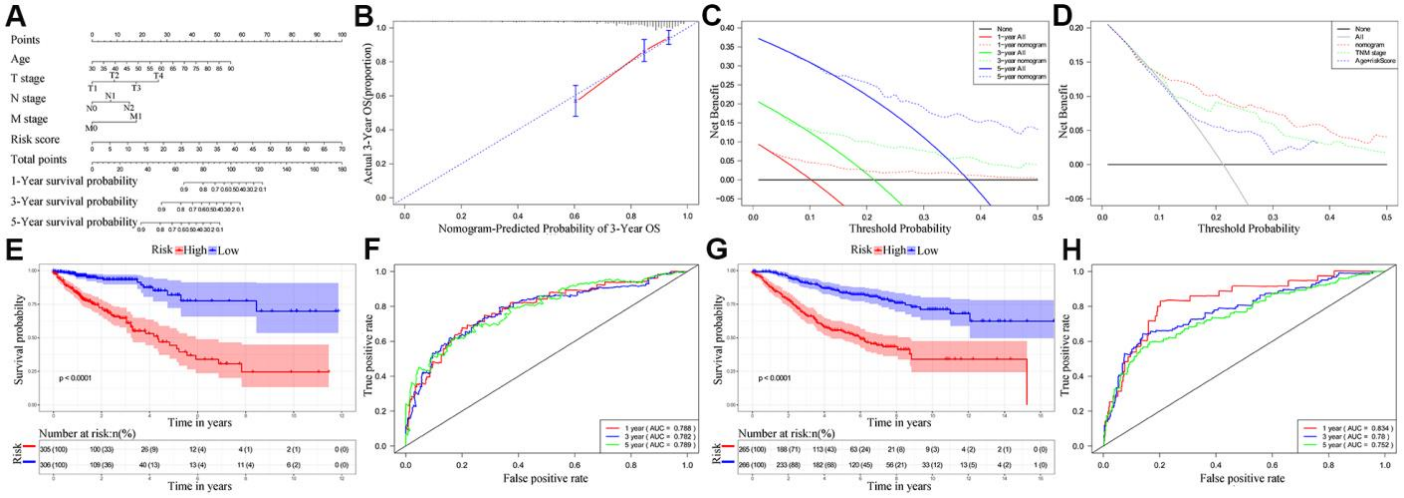
**Establishment and validation of the nomogram for predicting overall survival of CRC patients in the TCGA and GSE39582 cohorts.** (**A**) Nomogram with the ten-IRG prognostic risk score, TNM stages and age for predicting the 1-year, 3-year, and 5-year OS of CRC patients. In the nomogram, each variable is assigned a score. The sum of scores for all variables is used to predict the probability of survival of the CRC patients. (**B**) Calibration plot shows the comparison between nomogram predicted and actual 3-year OS of the TCGA cohort. (**C**) Decision curve analysis shows the predicted 1-year, 3-year and 5-year overall survival of CRC patients based on the nomogram. (**D**) Decision curve analysis shows the predicted 1-year, 3-year, and 5-year OS of CRC patients based on the nomogram, TNM stage only, and age plus ten-IRG signature. (**E**) Kaplan–Meier survival curves show the overall survival of CRC patients in the TCGA cohort based on the nomogram. (**F**) Time-dependent ROC curves show the accuracy of overall survival prediction in the TCGA cohort based on the nomogram. (**G**) Kaplan–Meier survival curves show the overall survival of CRC patients in the GSE39582 cohort based on the nomogram. (**H**) Time-dependent ROC curves show the accuracy of overall survival prediction in the GSE39582 cohort based on the nomogram.

Next, we analyzed if the ten-IRG prognostic signature would enhance the accuracy of predicting OS of CRC patients. Towards this, we constructed a nomogram with the ten-IRG signature-based risk score, M stage, N stage, T stage, and age to determine the 1-, 3-, and 5-year OS of CRC patients (Figure 6A). The calibration plots showed that the nomogram accurately predicted the 3-year OS of CRC patients (Figure 6B). Overall, the nomogram significantly improved the prediction of the 1-, 3-, and 5-year OS of CRC patients (Figure 6C). The OS prediction of the nomogram was significantly higher compared to the OS prediction based on TNM stages alone or age plus the ten-IRG signature-based risk score (Figure 6D). The C-index value for the nomogram was 0.769 (95% CI, 0.717-0.821). The AUC values for the 1-, 3-, and 5-year OS based on the nomogram were 0.788, 0.782 and 0.789, respectively (Figure 6F). We then verified the predictive value of the nomogram in the TCGA and GSE39582 validation cohorts. The C-index value for the internal validation cohort (TCGA) was 0.781 (95% CI, 0.709-0.853), and the AUC values for 1-, 3-, and 5-year OS were 0.834, 0.780, and 0.752, respectively (Supplementary Figure 2). The C-index value for the GSE39582 cohort was 0.732 (95% CI, 0.691-0.773) and the AUC values for 1-, 3-, and 5-year OS were 0.834, 0.780, and 0.752, respectively (Figure 6H). Furthermore, we stratified the CRC patients into high- and low-risk groups based on the median risk score cut off value of the nomogram. Kaplan-Meier survival curves and time-dependent ROC curves showed significantly worse survival outcomes for patients from the high-risk group compared to those from the low-risk group in both the TCGA cohort (P<0.001; Figure 6E, 6F) and GSE39582 cohort (P<0.001; Figure 6G, 6H and Supplementary Figure 2).

### Survival and immune cells infiltration analysis of ten IRGs in signature

**Figure 7:**
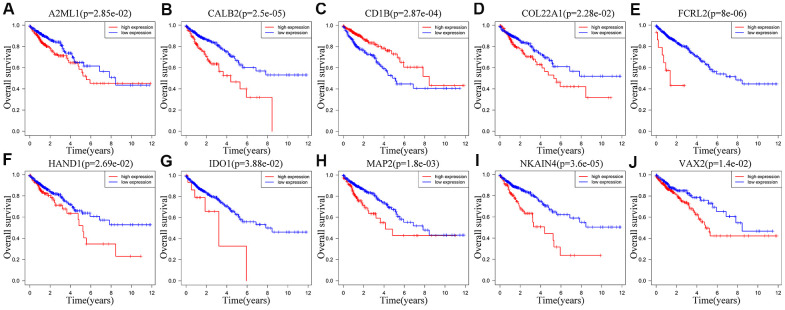
**Correlation between the expression levels of individual IRGs from the ten-IRG prognostic signature and OS of CRC patients in the TCGA database**. Kaplan-Meier survival plots show OS of CRC patients with high (red line) and low (blue line) expression of the ten individual IRGs. Kaplan-Meier survival curves for (**A**) A2ML1, (**B**) CALB2, (**C**) CD1B, (**D**) COL22A1, (**E**) FCRL2, (**F**) HAND1, (**G**) IDO1, (**H**) MAP2, (**I**) NKAIN4, (**J**) VAX2. Note: p<0.05 by the log-rank test.

We performed Kaplan-Meier survival curve analysis of the TCGA-CRC patients based on the expression of the ten prognostic IRGs to determine their independent prognostic values. High expression of A2ML1 (Figure 7A), CALB2 (Figure 7B), COL22A1 (Figure 7D), FCRL2 (Figure 7E), HAND1 (Figure 7F), IDO1 (cut off value: 34, Figure 7G), MAP2 (Figure 7H), NKAIN4 (Figure 7I) and VAX2 (Figure 7J) correlated with shorter OS in CRC patients, whereas, high expression of CD1B (Figure 7C) significantly correlated with longer OS. We observed similar results in the GSE39582 cohort (Supplementary Figure 3A–3J). Interestingly, IDO1 may also have another tumor suppressor gene identity in the GSE39582 cohort (Supplementary Figure 3G) and in TCGA database when expression was between 1.06 and 34 (Supplementary Figure 4).

**Figure 8:**
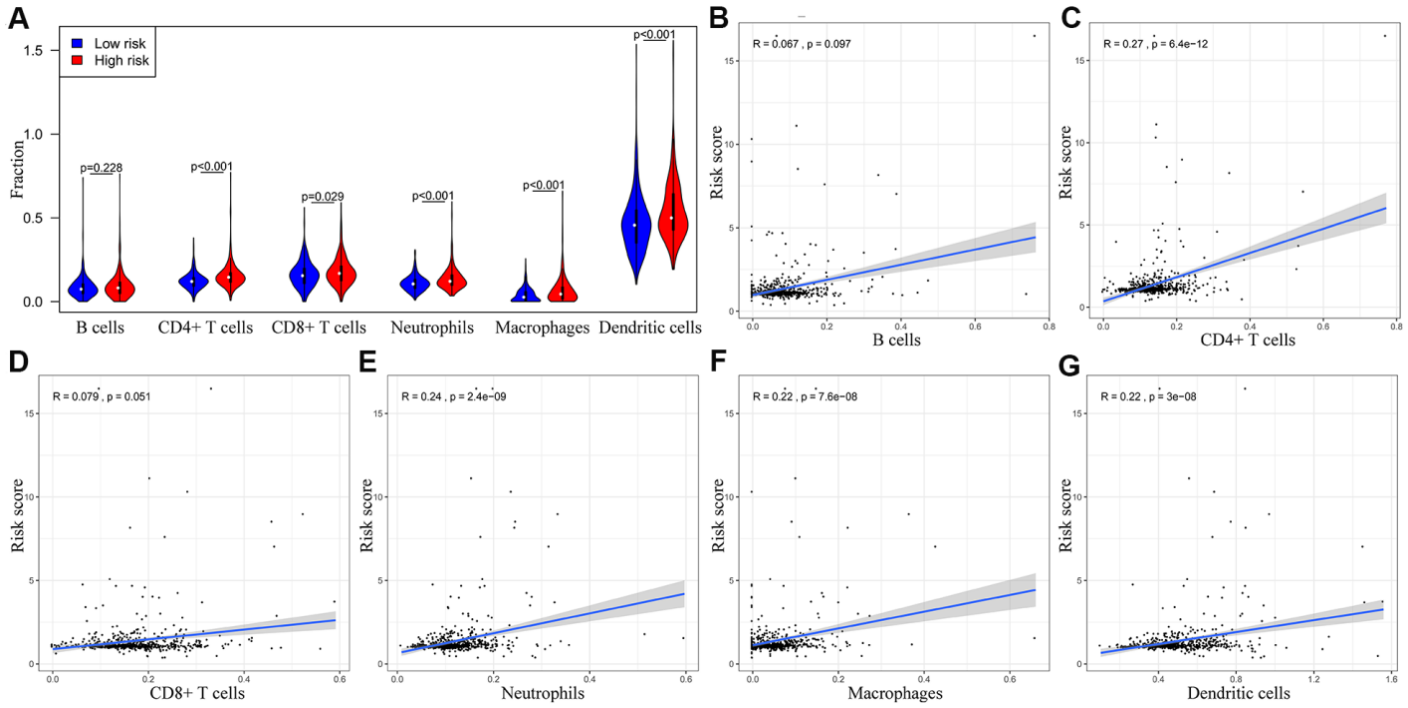
**The abundance of six immune cell types in the CRC tissues correlates with the risk scores according to the ten-IRG prognostic signature.** (**A**) The abundance of six immune cell types (B cells, CD4+ T cells, CD8+ T cells, macrophages, neutrophils, and dendritic cells) in the high and low risk groups based on the ten-IRG prognostic signature. (**B**–**G**) The correlation between the ten-IRG prognostic signature and the abundance of B cells, CD4^+^ T cells, CD8^+^ T cells, macrophages, neutrophils, and dendritic cells in the CRC tissues.

**Figure 9:**
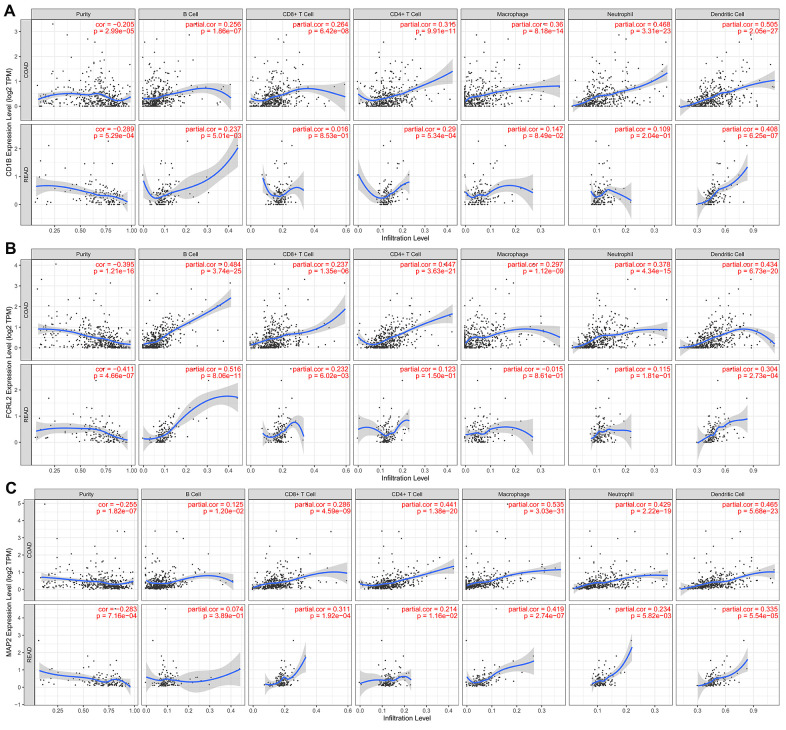
**The expression of specific prognostic IRGs correlates with the abundance of tumor-infiltrated immune cell types in the CRC tissues.** (**A**) The correlation analysis between *CD1B* gene expression and the proportions of six types of immune cells (B cells, CD4^+^ T cells, CD8^+^ T cells, macrophages, neutrophils, and dendritic cells) in the CRC tissues. (**B**) The correlation analysis between *FCRL2* gene expression and the proportions of six types of immune cells (B cells, CD4^+^ T cells, CD8^+^ T cells, macrophages, neutrophils, and dendritic cells) in the CRC tissues. (**C**) The correlation analysis between *MAP2* gene expression and the proportions of six types of immune cells (B cells, CD4^+^ T cells, CD8^+^ T cells, macrophages, neutrophils, and dendritic cells) in the CRC tissues.

We performed TIMER database analysis to determine the correlation between the ten-IRG prognostic signature and tumor infiltration of six types of immune cells, namely B cells, CD4^+^ T cells, CD8^+^ T cells, macrophages, neutrophils, and dendritic cells in the TME. The ten-IRG signature-related risk score showed positive correlation with the levels of CD4^+^ T cells, CD8^+^ T cells, macrophages, neutrophils, and dendritic cells in the TME (all P < 0.05), but was not significantly associated with the levels of B cells (P > 0.05; Figure 8). The levels of CD1B expression correlated with the infiltration levels of neutrophils (r= 0.468, p= 3.31E-23) and dendritic cells (r = 0.505, P = 2.05E-27) in colon adenocarcinoma (COAD) samples (Figure 9A and Table 1). Moreover, FCRL2 expression levels showed significant correlation with the infiltration levels of B cells (r= 0.484, p= 3.74E-25), CD4^+^ T Cells (r= 0.447, p= 3.63E-21), and dendritic cells (r = 0.434, P = 6.73E-20) (Figure 9B and Table 1). IDO1 expression levels were significantly associated with the infiltration levels of CD8^+^ T cells (r = 0.4, P = 4.66E-17), neutrophils (r= 0.638, p= 3.97E-47), and dendritic cells (r = 0.564, P = 3.47E-35) (Table 1). MAP2 expression levels were significantly associated with the infiltration levels of CD4^+^ T Cells (r= 0.441, p= 1.38E-20), macrophages (r= 0.535, p= 3.03E-31), and dendritic cells (r = 0.465, P = 5.68E-23) (Figure 9C and Table 1).

**Table 1:** Correlation analysis between the ten prognostic IRGs and immune cell infiltration in the CRC tissues.

**IRGs**	**CRC**	**Tumor purity**		**B Cells**		**CD8^+^ T Cells**		**CD4^+^ T Cells**		**Macrophages**		**Neutrophils**		**Dendritic cells**	
	**TYPES**	**Cor**	**P**	**Cor**	**P**	**Cor**	**P**	**Cor**	**P**	**Cor**	**P**	**Cor**	**P**	**Cor**	**P**
**A2ML1**	COAD	-0.156	0.002	-0.103	0.039	-0.016	0.743	0.136	0.006	0.048	0.339	0.019	0.705	0.025	0.624
	READ	-0.029	0.73	-0.04	0.639	-0.119	0.162	-0.101	0.239	0.014	0.866	-0.005	0.957	0.115	0.179
**CALB2**	COAD	-0.291	2.26E-09	0.014	0.769	0.109	0.028	0.263	9.11E-08	0.331	8.51E-12	0.284	7.19E-09	0.299	8.76E-10
	READ	-0.334	5.49E-05	-0.081	0.345	-0.002	0.984	0.286	6.37E-04	0.321	1.16E-04	0.204	0.02	0.291	5.13E-04
**CD1B**	COAD	-0.205	2.99E-05	0.256	1.86E-07	0.264	6.42E-08	0.315	9.91E-11	0.36	8.18E-14	0.468	3.31E-23	0.505	2.05E-27
	READ	-0.289	5.29E-04	0.237	0.005	0.016	0.853	0.29	5.34E-04	0.147	0.085	0.109	0.204	0.408	6.25E-07
**COL22A1**	COAD	-0.265	5.40E-08	0.022	0.664	0.131	0.008	0.396	1.62E-16	0.473	6.29E-24	0.398	1.06E-16	0.395	1.91E-16
	READ	-0.296	3.84E-04	0.099	0.242	0.0006	0.994	0.411	5.07E-07	0.399	1.16E-06	0.254	0.002	0.299	3.50E-04
**FCRL2**	COAD	-0.395	1.21E-16	0.484	3.74E-25	0.237	1.35E-06	0.447	3.63E-21	0.297	1.12E-09	0.378	4.34E-15	0.434	6.73E-20
	READ	-0.411	4.66E-07	0.516	8.06E-11	0.232	0.006	0.123	0.15	-0.015	0.861	0.115	0.181	0.304	2.73E-04
**HAND1**	COAD	-0.176	3.49E-04	-0.103	0.038	0.038	0.446	0.256	2.06E-07	0.377	4.04E-15	0.155	0.002	0.211	1.93E-05
	READ	-0.067	0.433	-0.016	0.856	-0.06	0.479	0.219	0.01	0.317	1.46E-04	-0.019	0.828	0.111	0.191
**IDO1**	COAD	-0.353	2.17E-13	0.248	4.42E-07	0.4	4.66E-17	0.286	5.44E-09	0.303	5.33E-10	0.638	3.97E-47	0.564	3.47E-35
	READ	-0.377	4.46E-06	0.25	0.003	0.342	3.82E-05	0.184	0.031	0.046	0.594	0.364	1.16E-05	0.567	3.38E-13
**MAP2**	COAD	-0.255	1.82E-07	0.125	0.012	0.286	4.59E-09	0.441	1.38E-20	0.535	3.03E-31	0.429	2.22E-19	0.465	5.68E-23
	READ	-0.283	7.15E-04	0.074	0.389	0.311	1.92E-04	0.214	0.012	0.419	2.74E-07	0.234	0.006	0.335	5.54E-05
**NKAIN4**	COAD	-0.274	1.92E-08	-0.07	0.161	-0.007	0.887	0.301	7.29E-10	0.341	1.90E-12	0.229	3.78E-06	0.273	2.79E-08
	READ	-0.325	9.17E-05	-0.08	0.327	-0.123	0.149	0.389	2.20E-06	0.454	1.93E-08	0.113	0.188	0.197	0.02
**VAX2**	COAD	-0.239	1.12E-06	-0.016	0.742	0.118	0.017	0.239	1.21E-06	0.352	3.26E-13	0.284	7.00E-09	0.285	5.61E-09
	READ	-0.108	0.203	-0.157	0.07	-0.119	0.163	0.206	0.015	0.139	0.104	0.012	0.89	0.096	0.261

## DISCUSSION

In this study, we used the ESTIMATE algorithm to analyze the transcriptome and clinical data of CRC patients from the GEO and TCGA databases to determine immune-related gene (IRG) signatures that predict survival outcomes. The ESTIMATE algorithm calculates stromal and immune scores and predicts tumor purity based on single-sample gene set enrichment analysis (ssGSEA) of specific gene signatures that are related to the proportions of stromal and immune cells in the TME. The accuracy of the ESTIMATE algorithm has been validated in several cancer types and shown promising results. However, the ESTIMATE algorithm has not been used to evaluate prostate or pancreatic cancers because of the atypical nature of the tumor cells and lack of sufficient data [16]. We screened differentially expressed IRGs by comparing CRC patients from the TCGA database with high and low immune, stromal or tumor purity scores and built a risk signature including ten IRG genes based on the LASSO Cox regression analyses. The ten-IRG risk signature was significantly associated with the OS of CRC patients from the TCGA and GSE39582 cohorts. Furthermore, the risk signature was significantly associated with the TNM stages. These results demonstrate that the ten-IRG signature predicts the prognosis of patients with CRC.

We then established a nomogram that included the risk score based on the ten-IRG signature and clinicopathological parameters including TNM stages and age to accurately predict OS of CRC patients. The predictive performance of the nomogram was satisfactory. The calibration plots demonstrated that the actual OS and predicted OS values were comparable. The C-index values for the nomogram were 0.769 and 0.732 for the TCGA and GSE39582 cohorts, respectively. The prognostic accuracy was higher when the clinicopathological parameters were combined with the ten-IRG prognostic signature compared to clinicopathological parameters alone or the ten-IRG prognostic signature alone. This demonstrated that the ten-IRG signature improved the prognostic prediction of the clinicopathological parameters.

In general, the status and function of tumor-infiltrating immune cells regulates the biological behavior of cancers. CD4^+^ and CD8^+^ T cells identify cancer cell-related antigens and play a significant role in cancer immunotherapy [20, 21]. Conversely, tumor-associated macrophages (TAMs) provide trophic and nutritional support to the malignant cancer cells and mediate therapeutic resistance and disease progression [22]. TAMs also represent potential targets for human anticancer therapies [23]. Neutrophils play a significant role in tumor initiation, growth and progression, and have been identified as therapeutic targets and clinical biomarkers in several cancers [24]. TIMER database analysis demonstrated significant positive correlation between the ten-IRG signature and the infiltration of CD4^+^ T cells, CD8^+^ T cells, macrophages, neutrophils, and dendritic cells. This suggests that higher tumor infiltration of immune cell types indicates CRC patients with advanced stage cancer. Furthermore, these data confirm the regulatory role of tumor-infiltrated immune cells in CRC progression.

We observed significant correlation between the expression of several genes in the ten-IRG signature and tumor progression. Bagchi et al. demonstrated the anti-tumor potential of CD1b-autoreactive T cells and their potential in adoptive immunotherapy [25]. Guo et al. demonstrated that miR-582/CD1B regulates the function of dendritic cells and is associated with immunotherapeutic outcomes in patients with lung adenocarcinoma [26]. The role of CD1B in CRC is unclear. Chronic inflammation is a well-established risk factor for CRC [27]. IDO1 plays an important role in limiting adaptive immune responses in several inflammatory and malignant gut diseases including gastric, pancreatic, esophageal, and stromal tumors [28]. High IDO1 expression is associated with tumor progression and poor clinical outcomes in CRC patients [29]. IDO1 contributes to immune tolerance in colon cancer by suppressing CD8^+^ T cell responses [30]. Reversing IDO1-mediated immunosuppression improved responses to immunogenic chemotherapy in a subcutaneous colorectal tumor model by promoting dendritic cell maturation, increasing tumor infiltrating T lymphocytes, and decreasing the numbers of regulatory T cells [31]. Our study showed IDO1 may be a gene with Janus face. On one face, IDO1 expression was higher than 34 that will be as an oncogene (Figure 7). The other face, IDO1 will be a cancer suppressor gene when expression was between 1.06 and 34 (Supplementary Figure 4). And the significant correlation was confirmed between IDO1 expression levels and the numbers of tumor-infiltrating dendritic cells and CD8^+^ T cells.

There are several limitations in our study. Firstly, we performed this study by analyzing data of CRC patients from public databases with genetic algorithms. However, the findings of our study require further clinical validation. Secondly, we did not validate the selected IRGs independently. Therefore, further studies are necessary to establish the individual role of these IRGs in tumorigenesis and immunotherapy response in CRC.

In conclusion, we used the ESTIMATE algorithm to analyze clinical and transcriptome data of CRC patients from the TCGA and GEO databases and identified a ten-IRG signature that is significantly associated with the status of tumor-infiltrating immune cells and prognosis of CRC patients. Further investigations are necessary to determine the clinical utility of this ten-IRG prognostic signature.

## MATERIALS AND METHODS

### Data collection

We searched the GSE and TGCA databases to identify CRC datasets with sample sizes exceeding 50 subjects. The selection criteria included: (1) RNA-Seq or microarray data from transcriptome studies; and (2) clinical data including data regarding clinicopathological parameters such as TNM stages and OS data. Based on these parameters, we selected six datasets, namely GSE103479, GSE72970, GSE41258, GSE39582, GSE12945, and TCGA [32–37] for this study. This included 1635 primary CRC patients that were staged according to the American Joint Committee on Cancer (AJCC) staging system. The clinicopathological data was downloaded from the GEO (https://www.ncbi.nlm.nih.gov/geo/) and TCGA (https://tcga-data.nci.nih.gov/tcga/) databases. The ESTIMATE algorithm was used to evaluate tumor purity as well as stromal and immune scores [16]. The entire TCGA dataset was used for genomic analysis and 60% of the patients from this dataset were randomly selected as an internal validation cohort. The results were also validated using the largest CRC dataset GSE39582 as the external validation cohort. This study complied with the approved publication guidelines for the TCGA and GEO databases. Since we used the data from these public databases, we did not require the approval of the ethics committee from our university or the consent of the patients included in this study.

### Identification of immune-related genes (IRGs)

The ESTIMATE algorithm was used to calculate tumor purity, stromal scores, and immune scores of the CRC patient samples, which were then classified into low and high groups using X-tile [38]. X-tile is a novel tool for assessing the biological relationships between potential biomarker genes and prognosis, and identifies optimal cut-points based on the gene expression values [38]. Then, we used the limma R package [39] to identify differentially expressed genes using the cutoff values set at false discovery rate (FDR) < 0.05 and log_2_ | fold change | > 1. Finally, we identified the prognostic immune-related genes (IRGs) by comparing their stromal scores, immune scores, and tumor purity.

### Functional enrichment analysis of IRGs

We used the clusterProfiler R package [40] to perform Gene Ontology (GO) and Kyoto Encyclopedia of Genes and Genomes (KEGG) pathway enrichment analysis and identified the most enriched biological pathways and functions related to the IRGs. We used the false discovery rate (FDR) to determine the adjusted P values and set the statistical significance as FDR < 0.05 to identify the most enriched GO terms for the biological processes (BP), molecular functions (MF), and cellular components (CC) as well as the KEGG pathways related to the IRGs.

### Establishment of the IRG prognostic signature for CRC

We investigated the association between IRGs and prognosis of CRC patients by performing univariate Cox regression analysis. P < 0.05 was considered statistically significant. Based on this analysis, we selected a panel of IRGs, which were then evaluated using the LASSO Cox regression analysis with the R package “glmnet”. We then set up a multi-gene signature to evaluate the prognosis of the CRC patients after cross-validating 1000 times with standard error within one standard deviation of the minimum. We then established the most simplified (smallest perimeter) immune gene expression signature model and calculated the risk scores for all patients based on the sum of the corresponding coefficients and expression value of each gene in the prognostic IRG signature model.

### TIMER database analysis

We used the deconvolution algorithm from the TIMER database (https://cistrome.shinyapps.io/timer/) to analyze the association between the prognostic risk-related IRGs and the tumor-infiltration levels of immune cells, such as B cells, CD4^+^ T cells, CD8^+^ T cells, macrophages, neutrophils, and dendritic cells [41].

### Statistical analysis

The median risk score was used as the cutoff value to classify CRC patients from the GSE39582 and TCGA datasets into low- and high-risk groups. Kaplan-Meier survival curves and log-rank test were used to evaluate the differences in OS rates between the low- and high-risk groups. Univariate and multivariate analysis of clinicopathological parameters and the ten-IRG prognostic signature was performed to determine the prognostic factors for the CRC patients from the TCGA dataset. A nomogram was constructed with the prognostic clinicopathological factors to determine the OS of the CRC patients in the TCGA cohort. The performance of the nomogram was evaluated using C-index and calibration plots with the “rms” R package. The efficiency of the nomogram was validated with the internal validation TCGA cohort and the GSE39582 cohort. Two-tailed t-tests were used to determine statistical significance, which was set at P < 0.05. R software version 3.6.0 was used to perform all statistical analyses.

## Supplementary Material

Supplementary Figures

Supplementary Table 1
