# Supplementary material for: Comprehensive analysis of prognostic immune-related genes in the tumor microenvironment of colorectal cancer
Source: Aging (Albany NY). 2021 Feb 1;13(4):5506–24. doi: 10.18632/aging.202479 (PMC7950244; doi:10.18632/aging.202479)
Supplement: Supplementary Figures [file aging-13-202479-s001.pdf]

SUPPLEMENTARY FIGURES

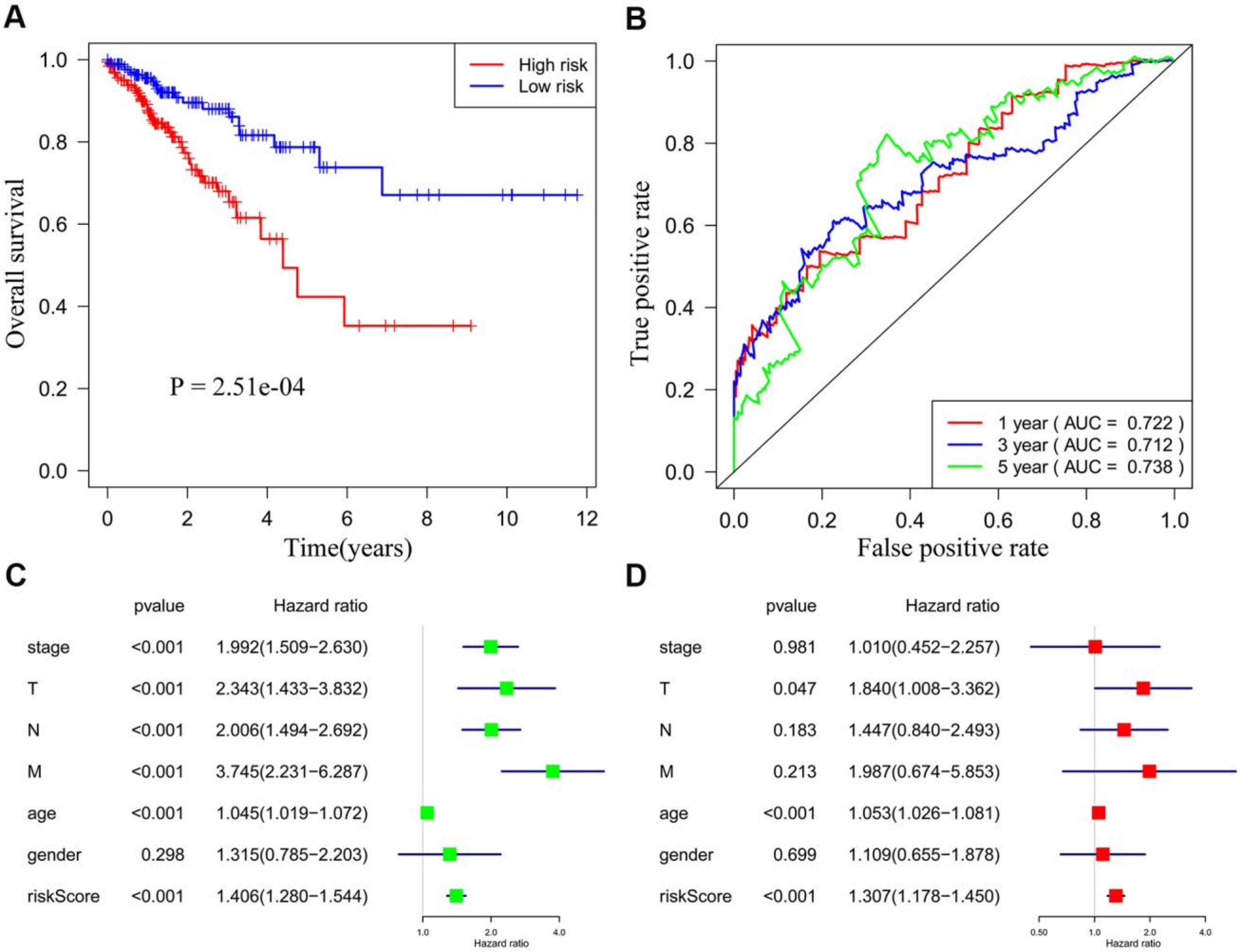

Supplementary Figure 1. Validation of the ten-IRG signature in the internal validation cohort. (**A**) Kaplan–Meier survival curves show the overall survival of high- and low-risk CRC patients in the internal validation cohort based on the ten-IRG prognostic signature. (**B**) Time-dependent ROC curves show accuracy of the overall survival prediction based on the ten-IRG prognostic signature in the internal validation cohort. (**C**) Univariate Cox regression analysis shows the clinicopathological parameters associated with overall survival of CRC patients in the internal validation cohort. (**D**) Multivariate Cox regression analysis shows the clinicopathological parameters associated with overall survival of CRC patients in the internal validation cohort.

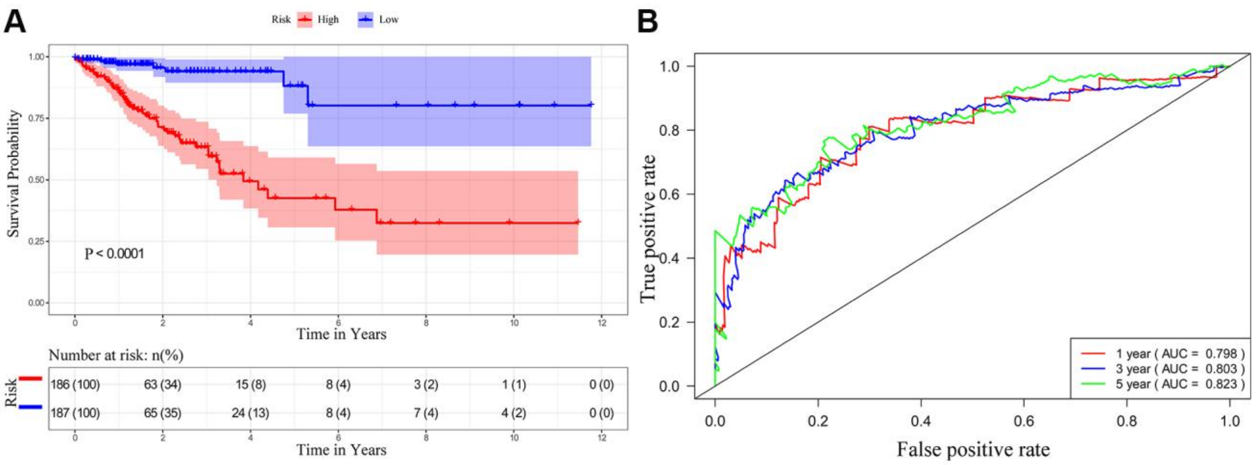

Supplementary Figure 2. Validation of the nomogram in accurately predicting overall survival of CRC patients in the internal validation cohort. (**A**) Kaplan–Meier survival curves show the overall survival of high-and low-risk CRC patients in the internal validation cohort based on the nomogram. (**B**) Time-dependent ROC curves show the accuracy of overall survival prediction based on the nomogram in CRC patients from the internal validation cohort.

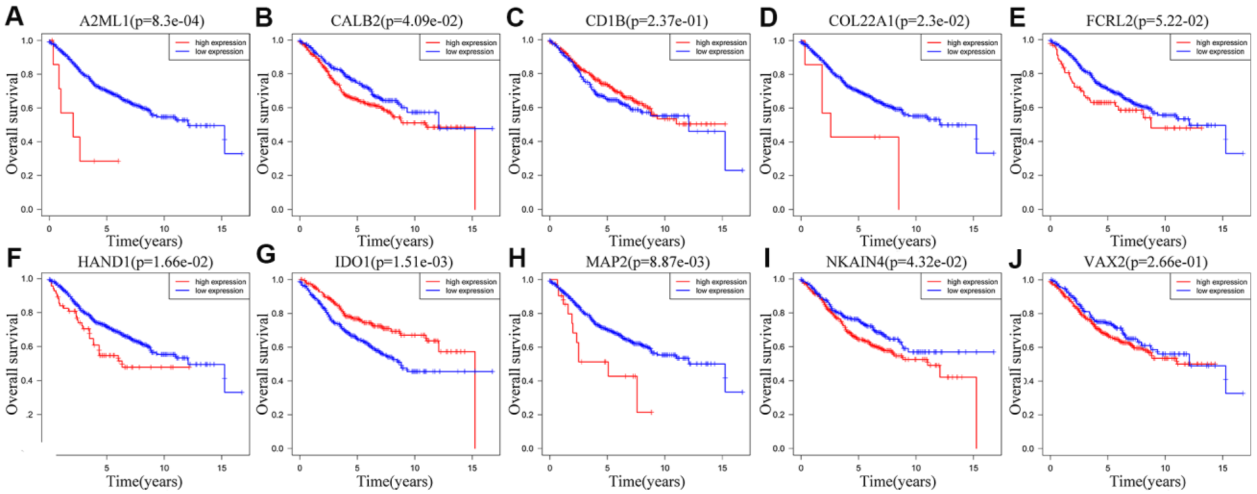

Supplementary Figure 3. Correlation analysis between expression levels of individual prognostic IRGs and OS of CRC patients in the GSE39582 cohort. Kaplan-Meier survival plots show the OS of CRC patients in the GSE39582 cohort with high (red line) and low (blue line) expression of the ten individual IRGs. Note: p<0.05 according to the log-rank test.

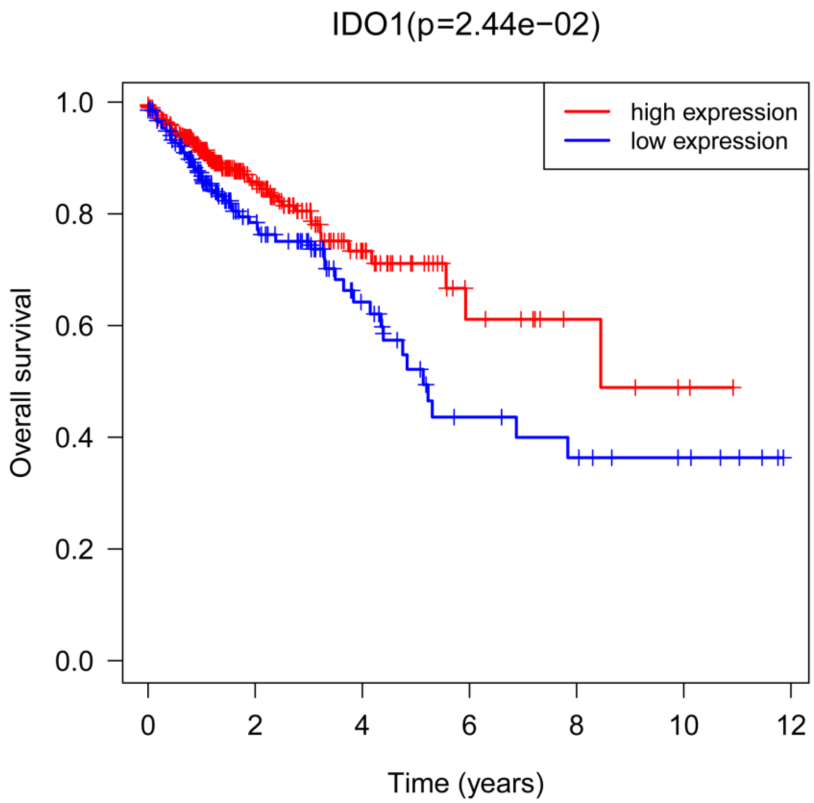

Supplementary Figure 4. Kaplan-Meier survival plot of IDO1 (cut-off value=1.06).
