# Supplementary material for: Comprehensive analysis of prognostic immune-related genes in the tumor microenvironment of colorectal cancer
Source: Aging (Albany NY). 2021 Feb 1;13(4):5506–24. doi: 10.18632/aging.202479 (PMC7950244; doi:10.18632/aging.202479)
Supplement: Supplementary Table 1 [file aging-13-202479-s002.pdf]

SUPPLEMENTARY TABLE

Supplementary Table 1. Top 20 KEEG pathways and GO terms enriched by the IRGs.

Categories	Term	Description	Count	FDR
**KEGG pathways**	hsa04060	Cytokine-cytokine receptor interaction	68	4.11E-21
	hsa04062	Chemokine signaling pathway	45	1.51E-14
	hsa04151	PI3K-Akt signaling pathway	42	0.000122
	hsa04145	Phagosome	41	3.35E-15
	hsa04061	Viral protein interaction with cytokine and cytokine receptor	40	3.85E-21
	hsa05152	Tuberculosis	39	2.31E-11
	hsa05150	Staphylococcus aureus infection	38	2.70E-20
	hsa04640	Hematopoietic cell lineage	38	7.47E-20
	hsa04514	Cell adhesion molecules (CAMs)	37	9.65E-13
	hsa04380	Osteoclast differentiation	35	3.21E-13
	hsa05323	Rheumatoid arthritis	33	5.13E-16
	hsa05140	Leishmaniasis	26	2.94E-12
	hsa05322	Systemic lupus erythematosus	26	1.23E-06
	hsa05166	Human T-cell leukemia virus 1 infection	24	0.01797
	hsa04610	Complement and coagulation cascades	23	1.06E-08
	hsa04620	Toll-like receptor signaling pathway	23	5.17E-07
	hsa05164	Influenza A	23	0.001474
	hsa04510	Focal adhesion	23	0.012233
	hsa04015	Rap1 signaling pathway	23	0.020275
	hsa05144	Malaria	22	4.66E-13
**GO BP**	GO:0002694	regulation of leukocyte activation	100	2.45E-33
	GO:0050900	leukocyte migration	98	1.54E-33
	GO:0042110	T cell activation	86	3.07E-27
	GO:0002521	leukocyte differentiation	86	1.34E-24
	GO:0001819	positive regulation of cytokine production	85	1.89E-26
	GO:0042119	neutrophil activation	85	3.42E-23
	GO:0043062	extracellular structure organization	83	3.31E-29
	GO:0050727	regulation of inflammatory response	82	1.68E-22
	GO:0002446	neutrophil mediated immunity	82	2.31E-21
	GO:0002283	neutrophil activation involved in immune response	81	2.31E-21
	GO:0043312	neutrophil degranulation	80	5.85E-21
	GO:0030198	extracellular matrix organization	78	7.27E-30
	GO:0051249	regulation of lymphocyte activation	77	3.24E-24
	GO:0050867	positive regulation of cell activation	75	4.78E-29
	GO:0032103	positive regulation of response to external stimulus	73	7.40E-28
	GO:0002683	negative regulation of immune system process	72	4.76E-18
	GO:0007159	leukocyte cell-cell adhesion	71	2.26E-25
	GO:0002697	regulation of immune effector process	71	5.28E-18
	GO:0002696	positive regulation of leukocyte activation	70	1.60E-26
	GO:0022407	regulation of cell-cell adhesion	70	2.12E-20
**GO CC**	GO:0031012	extracellular matrix	107	1.76E-41
	GO:0098552	side of membrane	62	3.05E-17
	GO:0030667	secretory granule membrane	60	3.28E-20
	GO:0098797	plasma membrane protein complex	49	1.32E-06
	GO:0009897	external side of plasma membrane	48	2.95E-19

	GO:0070820	tertiary granule	40	2.38E-16
	GO:0005788	endoplasmic reticulum lumen	39	5.17E-07
	GO:0045121	membrane raft	38	1.69E-06
	GO:0098857	membrane microdomain	38	1.74E-06
	GO:0098589	membrane region	38	3.53E-06
	GO:0043025	neuronal cell body	38	0.004156
	GO:0030139	endocytic vesicle	37	9.93E-07
	GO:0060205	cytoplasmic vesicle lumen	37	2.55E-05
	GO:0031983	vesicle lumen	37	2.65E-05
	GO:0034774	secretory granule lumen	36	2.13E-05
	GO:0042581	specific granule	35	8.24E-13
	GO:0043235	receptor complex	35	7.29E-06
	GO:0031252	cell leading edge	32	0.009968
	GO:0005581	collagen trimer	31	7.80E-18
	GO:0005925	focal adhesion	31	0.027118
**GO MF**	GO:0030545	receptor regulator activity	60	3.55E-12
	GO:0048018	receptor ligand activity	59	7.02E-13
	GO:0030246	carbohydrate binding	54	1.28E-18
	GO:0005539	glycosaminoglycan binding	53	7.93E-23
	GO:0005201	extracellular matrix structural constituent	52	5.50E-27
	GO:1901681	sulfur compound binding	44	6.34E-14
	GO:0008201	heparin binding	41	2.33E-18
	GO:0005126	cytokine receptor binding	37	3.10E-10
	GO:0005125	cytokine activity	35	7.02E-13
	GO:0008047	enzyme activator activity	35	0.010079
	GO:0001664	G-protein coupled receptor binding	34	9.06E-09
	GO:0033218	amide binding	31	8.09E-05
	GO:0042277	peptide binding	29	1.07E-05
	GO:0004175	endopeptidase activity	29	0.008894
	GO:0003779	actin binding	29	0.037972
	GO:0005178	integrin binding	25	5.00E-09
	GO:0019955	cytokine binding	23	9.95E-09
	GO:0005518	collagen binding	21	2.15E-11
	GO:0019838	growth factor binding	21	6.77E-06
	GO:0031406	carboxylic acid binding	21	0.00145
